# The Identification of Polyester Fibers Dyed with Disperse Dyes for Forensic Purposes

**DOI:** 10.3390/molecules24030613

**Published:** 2019-02-10

**Authors:** Daria Śmigiel-Kamińska, Jan Pośpiech, Joanna Makowska, Piotr Stepnowski, Jolanta Wąs-Gubała, Jolanta Kumirska

**Affiliations:** 1 Faculty of Chemistry, University of Gdańsk, ul. Wita Stwosza 63, 80-308 Gdansk and Poland; pospiechjan@gmail.com (J.P.); joanna.makowska@ug.edu.pl (J.M.); piotr.stepnowski@ug.edu.pl (P.S.); 2 Institute of Forensic Research, Criminalistics Department, Westerplatte 9, 3-033 Krakow, Poland; jwas@ies.krakow.pl

**Keywords:** polyester fibers, dyed fibers, disperse dyes, forensic analysis, chromatographic methods, spectroscopic methods

## Abstract

In forensic laboratories, the most commonly analyzed microtraces are microscopic fragments of single fibers. One of the main goals of the examination of fragments of fibers a few millimeters long is to determine their characteristic physicochemical properties and compare them with fibers originating from a known source (e.g., a suspect’s clothes). The color and dyes of fiber microtraces play an important role in their research and evaluation, being analyzed by means of microscopic, spectroscopic, and chromatographic methods. The results of examinations conducted with the use of spectroscopic techniques might be ambiguous due to overlapping bands of absorption and the transmission and dispersion of electromagnetic radiation corresponding to the specific chemical structure of the fibers and their dyes. For this reason, it is very important to improve currently available spectroscopic methods and/or to propose new ones that allow evidential materials to be analyzed in a much more reliable way. In this review, the possibility of the use of chromatographic techniques with different detection systems for such analyses is underlined. This review covers the different analytical methods used in the forensic analysis of polyester fibers dyed with disperse dyes. Polyester fibers occupy the first position among synthetic fibers in their use for a variety of purposes, and disperse dyes are commonly applied for dyeing them.

## 1. Introduction

Fibers accompany us every day. Clothing, the upholstery of home and office furniture as well as that of cars, and biomedical materials are produced from fibers, therefore, they can act as a *mute witness* in a criminal case. Following studies on microtraces in the form of fragments of single fibers, experts from forensic laboratories are able to establish a relationship between individuals, objects, and a crime scene, and sometimes fibers can also help to reconstruct the circumstances of the event. This denotes that even the smallest fragment (e.g., fibers of approx. 0.5 mm in length with a cross-section of approx. 10 microns) may constitute evidence in a lawsuit. More and more frequently, the analysis of microtraces can explain incidents such as homicides, sexual offenses, robberies, and traffic accidents. The main goal of the identification and comparison of microtraces in the form of fragments of single fibers is to determine their physicochemical properties and then qualify them to a specific type of fiber and assortment of textile products. An important role in the first step of the comparison of such microtraces is played by their color. On the basis of color analysis, evidential fibers can be eligible for further study with a comparative material or they are discarded and considered as different from the comparative material.

Because dyes can provide significant information in forensic analyses, many studies have already been carried out to analyze dyes in colored fibers by using diverse microscopical and spectral methods, e.g.,: microspectrophotometry [1,2,3,4], Raman spectroscopy [1,5,6,7,8], excitation-emission luminescence [9], infrared matrix-assisted laser desorption electrospray [10]; as well as chromatography methods, e.g.,: thin liquid chromatography (TLC) [8,11,12], liquid chromatography (HPLC) with different detectors [12,13,14,15,16,17,18,19,20,21], and capillary electrophoresis with a DAD or MS detector [22,23,24,25,26,27,28].

Among man-made fibers, polyesters are produced in the greatest amounts (43,301 million metric tons; 75% of global man-made fiber production in 2012) [26], and disperse dyes are mostly used for dyeing them. For these reasons, single polyester fibers are very often subjected to analysis in forensic laboratories. Unfortunately, there are a limited number of chromatographic methods developed for this purpose [26,27,28]. There is also no review paper concerning this subject. For this reason, the main aims of this review are as follow: (1) to present the possibility of the application of chromatographic methods for the analysis of polyester fibers dyed with disperse dyes for forensic purposes; (2) to present the possibility of the application of spectroscopic methods for such analyses; (3) to compare a chromatographic approach with a spectroscopic one; (4) to present new challenges in identifying dyed fibers for forensic purposes.

## 2. Literature Review Sections

### 2.1. Disperse (Suspension) Dyes in the Textile Industry

Worldwide production of organic dyes is currently estimated at nearly 450,000 tons. Disperse dyes are low-molecular-weight synthetic organic dyes in the range of 400–600 that represent at least 22% of the dyes consumed in the world [29,30,31,32,33,34,35,36]. They are marketed in the form of either an easily dispersible powder or a concentrated aqueous dispersion. The Color Index listed around 1150 disperse dyes in 1992 by chemical class, i.e., azo, anthraquinone, meethine, nitrodiphenylamine, xanthene, aminoketone, quinoline, and miscellaneous [29]. Most disperse dyes are essentially planar compounds bearing an azo chromophore attached to polar functional groups such as amine and nitro, and halogens like chlorine or bromine. Color changes are caused by changes in the extent of the delocalization of electrons. More delocalization shifts the absorption max to longer wavelengths and makes the light absorbed redder, while less delocalization shifts the absorption max to shorter wavelengths [36].

Disperse (suspension) dyes are non-ionic substances, practically insoluble in water and in an aqueous solution, and exist in the form of well-dispersed suspension. Disperse dyes in the form of individual molecules penetrate into hydrophobic fibers. It should be noted that these types of dyes may diffuse into the free spaces of a fiber. Such a process is observed when the system exceeds the glass transition temperature. Disperse (suspension) dyes are used for dyeing hydrophobic fibers from cellulose acetate, polyester, and polyamide [29,30,31,32].

**Figure 1:**
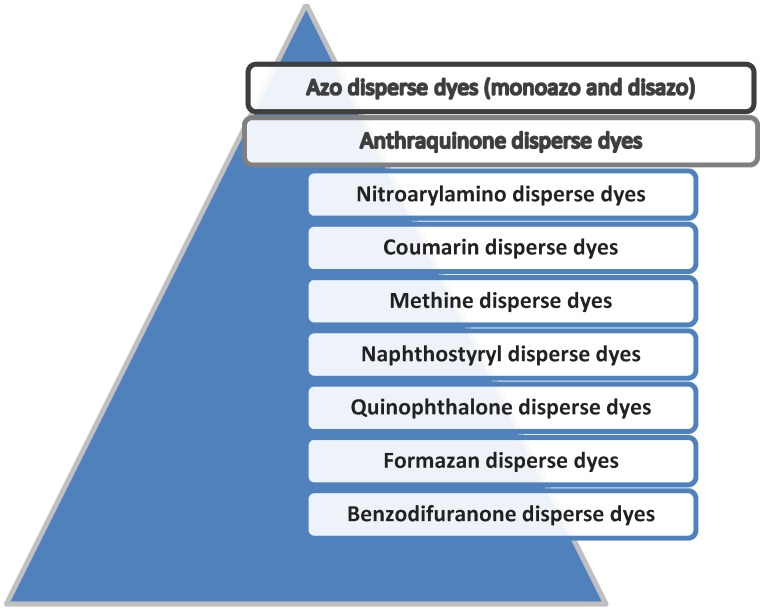
Classification of disperse dyes by chromogen.

The classification of disperse dyes by chromogen (Figure 1) is very useful not only for dye chemists but also for forensic analysts. As was mentioned, azo disperse dyes (monoazo and disazo) as well as anthraquinone disperse dyes are the most important classes of disperse dyes in terms of market share, and for this reason, the probability of them being a subject of forensic investigations is the highest.

### 2.2. Extraction of Disperse Dyes from Dyed Textile Fibers for Forensic Purposes

The extraction method depends directly on the dye class and fiber type [15]. Polyester fibers as well as disperse dyes are hydrophobic [21]. Thus, the best results of extraction should be obtained using solvents characterized by a low polarity, i.e., a small value of a dielectric constant. However, it is also very important to choose a solvent that will not make the experiment difficult at the later stage of the dye identification procedure [12].

Table S1, included in the Appendix, shows methods for the extraction of disperse dyes described in the literature [12,13,14,15,16,17,18,19,20,21]. All the studies concerned polyester fibers. A brief description of each extraction procedure shown in Table S1 is presented below.

Method No. 1—Eight different solvents were tested for the extraction of dyes from fibers; the best results were obtained using boiling chlorobenzene [12]. Fibers or small pieces of fabric were introduced into small beakers (5 mL) or test tubes (50 × 6 mm). Next, 0.5–3 mL of chlorobenzene was added and the samples were put on a hot plate or in an oil bath and boiled for 5 min or until most of the dye had been extracted [12].

Method No. 2—Two alternative extraction solvents were used: chlorobenzene and dimethylformamide (DMF). In the first case, a single 2–5 mm-long fiber was pushed to the bottom of a 5 cm length of glass capillary tubing and 5 µL of chlorobenzene was added. The tube with fibers was sealed and heated at 100 °C for 15 min. The extract was evaporated to dryness by heating at 100 °C for 30 min and the residue was dissolved in 5 µL of HPLC mobile phase [13]. In the second case, the fibers were introduced into tubes and extracted using 4 µL of a mixture of dimethylformamide (DMF) and acetonitrile (ACN) (1:1, *v*/*v*). The dimethylformamide had been previously modified by the addition of 1.25 g of 2,6-di-*tert*-butyl-4-methylphenyl and 1 g of citric acid to each 250 mL of solvent. The unsealed tubes were agitated by hand for 1–2 min in a silicone oil bath at 120–130 °C. After cooling, 6 µL of mobile phase was added to the samples [13].

Method No. 3—Nine disperse dyes were extracted from 10 cm or less single polyester fibers. The fibers were placed in a capillary tube and 5 µL of chlorobenzene was added. The sealed tube was heated in an oven to 130 °C for 30 min. After this time, the fiber was removed and the extract was evaporated to dryness. The dry residue was dissolved in 15 µL of acidified acetonitrile containing an internal standard at a concentration of 1 ng per 5 µL injection [14].

Method No. 4—A single fiber of 10 mm in length was subjected to extraction. The extraction was carried out for fiber discoloration but for not longer than 2 h [15].

Method No. 5—Single filaments of a length of 0.5 mm, 2 mm, and 5 mm were extracted in Waters Total Recovery^®^ liquid vials at 100 °C for 1 h. After extraction, the solvent was evaporated to dryness at 90 °C. The dried residue was dissolved in 50 μL of methanol (MeOH) and 50 mM ammonium acetate at pH 4.5 [16].

Method No. 6—During this study, nine disperse dyes were isolated from single polyester fibers (5 mm in length) or from polyester thread (7 mm in length). Based on diode array detector data, dimethylformamide (DMF) was found to be a more effective extraction solvent than acetonitrile/water (4:3, *v*/*v*) or methanol/water (1:1, *v*/*v*) mixtures [17].

Method No. 7—Standardized fibers of a length of 20 mm were extracted in plugged glass capillaries [18].

Method No. 8—0.5 g of cut polyester fabric was subjected to extraction using 5 mL of solvent; the procedure was repeated, and the obtained extracts combined. The extraction was carried out in glass tubes using an ultrasonic bath. The extracts were evaporated to dryness under a stream of nitrogen and then dissolved in 1 mL of methanol and filtered [19].

Method No. 9—Single fibers of 10 mm in length were extracted using 20 μL of dimethylsulfooxide (DMSO) at 100 °C to discoloration or for a maximum of 2 h [20].

Method No. 10—Single 0.5-cm polyester fibers were extracted in a sealed glass capillary using 20 µL of acetonitrile. Extraction was initiated using ultrasonication for 5 min, then the sample was heated in a water bath at 60 °C for 60 min. After centrifugation, the obtained extracts were ready for HPLC-MS/MS analysis [21].

**Figure 2:**
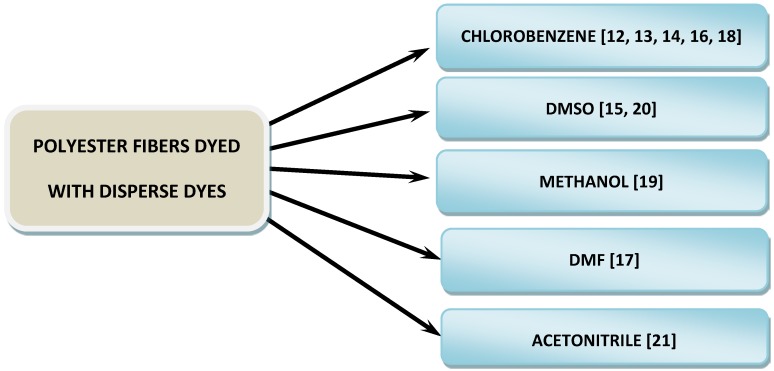
Solvents applied for the extraction of disperse dyes from dyed polyester fibers.

According to the presented above data [12,13,14,15,16,17,18,19,20,21], solvents commonly used for the extraction of disperse dyes from polyester fibers are as follow: chlorobenzene, methanol (MeOH), acetonitrile (ACN), dimethylsulfooxide (DMSO), and dimethylformamide (DMF) (Figure 2). The highest value of the dielectric constant of the presented solvents is for DMSO (~46.7) and the lowest for chlorobenzene (~9). The other remaining solvents, i.e., MeOH, can, and DMF, are similar, taking into account the polarity of the system (~<33–37>).

The most commonly used solvent for this purpose is chlorobenzene [12,13,14,16,18] (Figure 2; Table S1). Extraction was carried out for fibers or small pieces of fibers in the range of length 2–20 nm [12,13,14,16,18]. Depending on the fiber size and temperature of extraction, the required volume of chlorobenzene varied from 5 µL [13,14] to 0.5–3.0 mL [12].

The extractions were carried out at high temperatures: 100 °C [13,16]; 130 °C [14,18], or at the boiling point of chlorobenzene (t = 138 °C) [12]. The extraction time at 100 °C was both 15 min [13] and 60 min [16], at 130° 10 min [18] or 30 min [14], and at the boiling point of chlorobenzene, the process was continued for 5 min or until most of the dyes had been extracted [12].

DMSO [15,20], MeOH [19], DMF [17], and ACN [21] as pure solvents were also found to be suitable for extraction of disperse dyes from colored polyester fibers. Depending on the type of disperse dyes and the amount of material, time and temperature of extraction was different.

In the case of DMSO, 10 mm pieces of fiber were submerged in 20 μL of solvent and heated to 100 °C until the fiber was discolored or for a maximum of 2 h [15,20]. Only in the case of one dye named Disperse Blue 73, the time was slightly shorter than 2 h [15].

For experiments performed using MeOH as an extractant (5.0 mL of MeOH; 0.5 g cut fabric), the temperature must achieve max. 70 °C (this is the lower temperature in comparison to 100 °C for DMSO), and the process should be continued for 15 min and repeated in the same conditions [19]. A mixture MeOH/water (1:1, *v*/*v*) was not as effective as DMF for the extraction of disperse dyes from colored fibers [17].

For extraction using DMF, 30 µL of solvent was placed with a textile thread in a glass capillary tube and a sample was heated at 100 °C in a heat block [17].

As mentioned above, ACN could be used as a pure solvent [21] or as a component of the extraction mixture [17] of disperse dyes. Pure ACN was applied for the extraction of the 0.5 cm piece of polyester fiber [21], while a mixture of ACN:H_2_O (4:3, *v*/*v*) for the extraction of the length of fiber 5 mm or 7 mm (tread) [17]. In the case of a mixture of ACN:H_2_O, the sample was heated at 100 °C for 30 min, and in the case of pure solvent (ACN), the extraction was initiated using ultrasonication for 5 min followed by heating in a water bath at 60 °C.

In summary, there is no universal solvent for the extraction of disperse dyes from polyester fibers. However, due to chlorobenzene being used the most often for such extractions, this solvent should be the first one tested by analysts planning such an experiment, before any of the other above-mentioned solvents. Optimization of the extraction procedure is crucial for further identification of dyes.

### 2.3. Identification of Dyed Textile Fibers for Forensic Purposes Based on Chromatographic Techniques

**Figure 3:**
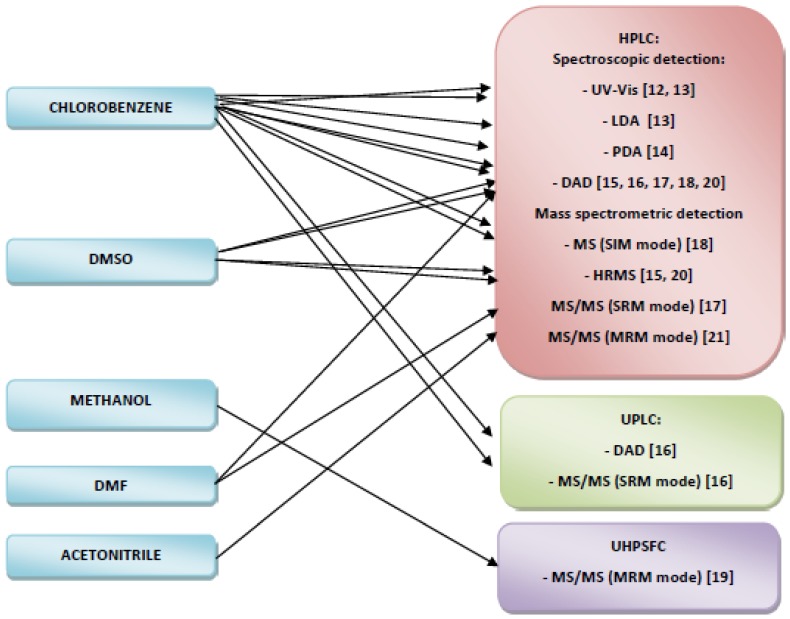
Chromatographic methods applied for the analysis of disperse dyes in extracts.

The methods currently used for forensic analyses should allow the analysis of individual fibers of 5 to 10 mm in length [18]. Such small amounts of the sample contain 2 to 200 ng of dye [18]. Therefore, analytical methods for identification must be sensitive enough to be able to detect such an amount of dye. Tables S2 and S3 included in the Supplementary show the methods developed for the identification of disperse dyes based on chromatographic techniques [12,13,14,15,16,17,18,19,20,21]. A description of the chromatographic conditions is included in Table S2 and the selected qualification and quantification parameters in Table S3. A general scheme of identification of disperse dyes extracted from colored polyester fibers for forensic purposes based on chromatographic techniques is presented in Figure 3.

The most commonly described chromatographic technique used for this purpose is high-performance liquid chromatography (HPLC) coupled with different types of spectrophotometric detectors: (UV-Vis) [12,13]; linear diode array (LDA) [13], photodiode-array (PDA) [14], diode array (DAD) [15,16,17,18,20], as well coupled with mass spectrometric (MS) [18], and high resolution mass spectrometric (HRMS) [15,20] and tandem mass spectrometric (MS/MS) detectors [17,21]. In many cases HPLC-MS apparatus contains both detection systems [15,17,18]. The most frequently used chromatographic column is the reverse-phase C18 column [13,18,21] whose length is from 50 mm to 1150 mm. Other types of chromatographic columns are: ODS-2 with length 150 mm [14,17] and ODS-5 with length 150 mm [15]. In one case, chromatographic analysis was performed using a LiCrosorb Si-60 column [12]. All chromatographic separation of disperse dyes was carried out under gradient conditions and with application of different mobile phases (water:MeOH with ammonium acetate [15,17,20], water:acetonitrile acidified [13,14], water:acetonitrile [18], acetic acid in water:acetic acid in acetonitrile [21], and hexane:ethyl acetate [12]). The time of analysis was from 20 min to 67 min. The mobile phase flow rate was from 0.2 mL/min to 1 mL/min with an injection volume of 5 μL or 10 μL.

Another chromatographic technique used for the identification of disperse dyes extracted from polyester fibers is ultra-performance liquid chromatography (UPLC) coupled with DAD (a wavelength range from 325 to 675 nm) and MS/MS detectors [16]. The separation was performed using a column C18 with length 50 mm. The analysis was carried out under gradient conditions; methanol with 0.15% formic acid and 50 mM ammonium acetate were used as the mobile phases. The time of analysis was 6.5 min, the mobile phase flow rate was from 0.6 mL/min and 0.3 mL/min (it was dependent on the type of detector), and the volume of injection was 10 μL [16].

Identification of disperse dyed extracted from polyester fibers was also performed using ultra-high-performance supercritical fluid chromatography coupled with tandem mass spectrometry technique (UHPSFC-MS/MS) [19]. In order to select the optimum stationary phase, four different chromatography columns (BEH, BEH2-ethylpyridine, HSSC18 SB and CSH fluorophenyl) were tested. The results showed that the best separation efficiency and the highest sensitivity were achieved using Waters ACQUITY UPC2 ™ BEH C18 columns. As mobile phases MeOH (A) and CO_2_ (B) were used. The time of analysis was 4.5 min. The flow rate was 2 mL/min, while the injection volume was 1 μL [19].

As mentioned, the chromatographic methods used for forensic analyses should allow the identification of individual fibers based on 2 to 200 ng of dye [18]. Therefore, knowledge about qualification and quantification parameters is also very important.

John C. West extracted and analyzed 18 disperse dyes on polyester textiles using TLC and HPLC (Tables S2 and S3, Method No. 1) [12]. Signals were registered at 420, 500, and 600 nm wavelengths. A comparison of both chromatographic approaches showed that the HPLC method gave slightly better resolution and better sensitivity, and consequently, smaller amounts of dyes could be examined and trace components could be easily observed. Moreover, poorly visible colors in TLC were visible in HPLC when the appropriate wavelength was chosen. Using the HPLC method, the differentiation of two dyes with the same R_f_ value in TLC was possible. Thus, the HPLC method was better than TLC [12].

The separated dyes could be monitored either using a single wavelength filter photometer or a multi-wavelength linear diode array detector (Tables S2 and S3) [13]. Identification of 57 disperse dyes extracted from dyed polyester fibers was based on retention parameters. Minimum detection levels of 200 pg of injected dye were obtained with both systems. These levels were 10–20 times those obtained with dye extracts originating from fibers of 2‒5 mm in length, although for very light shades the sensitivity was only just adequate. This method, based on the extraction of 2‒5 mm of single fibers, provided the discrimination of all fibers examined [13].

For the separation and characterization of acid, basic, and disperse dyes, Speers and co-workers used a gradient elution HPLC system with an end-capped narrow-bore reversed phase column and photodiode-array detection (Tables S2 and S3, Method No. 3) [14]. Standard commercial dyes were used for the optimization procedure. Spectral data of the standard commercial dyes (including nine disperse dyes) separated using the HPLC system, together with their relative retention times with respect to Rhodamine B, were stored in a library created using Millennium PDA software. Basic and disperse dyes extracted from 10 mm fibers were qualitatively and quantitatively identified using a database of relative retention times and the spectral data of standard commercial dyes. The system exhibited good reproducibility, efficiency, and sensitivity [14].

The identification of dyes extracted from fibers dyed with Disperse Blue 73 (Tables S2 and S3, Method 4) was based on retention times, and the mass accuracy (deviation generally < 2 ppm) was recorded using a high-resolution MS detector [15]. In addition, for each standard solution of dye and for each dye extracted from a fiber, the absorption spectrum was recorded using a DAD detector. The absorption spectra were compared using special software that determined the spectrum match index of a standard solution of dye with the spectrum of a dye extracted from the sample. If the index was more than 800, they considered the spectra to be quite similar, indicating that this is probably the same substance. If the index was above 900, the spectra were recognized as almost identical, ensuring that the standard and analyte are the same compound. The limits of detection (LODs) were shown as the minimum concentration of dissolved dye powder (μg/L) and the minimum fiber length required to identify the dye. The LOD values established using the DAD detector were 50.7 μg/L and 0.14 mm, while for the MS detector they were 1.1 μg/L and 0.003 mm, respectively [15].

Hoy, in his work [16], compared the results of identification and quantification of nine dyes, among them three belonging to the disperse class (Violet 77, Disperse Blue 60, Disperse Yellow 114) extracted from single fibers of 0.5, 1, and 5 mm in length (Tables S2 and S3, Method No. 5). Disperse Violet 77 and Disperse Yellow 114 dyes were detected and identified in all fibers while Disperse Blue 60 was detectable only in 1 and 5 mm fibers. Compared to other dye classes, the detection limits for disperse dyes were higher. The detection limit for these dyes ranged from 0.99 to 12.60 ppb for calibration concentrations from 10 to 50 ppb, except for Disperse Blue 60, for which it was 50 ppb. The DAD detector turned out to be more sensitive than MS-MS, probably due to the incorrect selection of ionization conditions [16].

Identification of nine disperse dyes isolated from polyester fibers (5 mm in length) was based on the retention times of isolated dyes and the selected reaction monitoring data (SRM) (Tables S2 and S3, Method No. 6). The LOD values were significantly lower than the amount of dye present in the protected fiber. The LOD values ranged from 1 to 15 pg for the linear ion trap tandem mass spectrometric detector (LIT-MS/MS) and from 750 to 1750 pg for the DAD detector [17].

The identification of dyes extracted from fibers from the upholstery of various car models was based, e.g., on the chromatograms and mass spectra (Tables S2 and S3, Method No. 7). Dorrien identified the Disperse Red 4 dye isolated from a standardized 20 mm fiber [18].

The identification of 17 disperse dyes isolated from the fabric samples was based on retention times and *m*/*z* values of parent/quantitative/assistant qualitative daughter ions (Tables S2 and S3, Method No. 8). The dye recovery from the fabric samples ranged from 70% to 103%. The detection limits were in the range of 0.02 to 1 μg/mL [19].

Schotman and co-workers developed Method No. 9 (Tables S2 and S3) for the analysis of dye in forensic fibers and textile examination on case examples [20]. Seven cases and a quality assurance test are presented. In these cases, the fibers or textiles submitted for investigation were analyzed using HPLC–DAD–MS to identify the dyes present. The results show that a mixture of dyes is present in all the textiles investigated except one sample that was taken from a manufacturer’s dye shade card. It is concluded that dye analysis improves the evidential value of forensic fiber examinations as it becomes possible to distinguish textiles that are different in dye chemistry but have a similar color. In addition, dye analysis makes the examination more robust as it becomes possible to attribute color differences between samples to identical dyes (mixed in different ratios) or to chemically different dyes [20].

The last method (Method No. 10, Tables S2 and S3), developed by Hu and co-workers [21], allowed a forensic fiber examination to be performed of 11 dyes representing five different classes of dyes, among them four disperse dyes (Disperse Yellow 9, Disperse Red 1, Disperse Violet 26 and Disperse Brilliant Red). Under the optimized conditions, the limit of detection (LOD) for disperse dyes reached 0.01–1.0 ng/mL in Multi Reaction Monitor (MRM) mode. This method allowed single fibers with a length of a few millimeters or less to be analyzed, which makes it suitable for forensic analysis.

Taking into account all the data presented above, it should be noticed that chromatographic methods have a great potential for the identification of polyester fibers dyed with disperse dyes for forensic purposes. Qualification and quantification parameters of such methods could be significantly improved by optimization of chromatographic conditions in HPLC/UPLC/UHPSFC systems and/or by application of more selective and sensitive detectors (UV-Vis < LDA < PDA < DAD, MS(SIM) < HRMS < MS/MS(SRM) < MS/MS(MRM). Extraction of disperse dyes from colored polyester fibers should also be performed in optimal conditions.

### 2.4. Identification of Dyed Textile Fibers for Forensic Purposes Based on Spectroscopic Techniques

During recent years, the application of spectroscopic techniques to the identification and discrimination of textile fibers has gradually increased. Microspectrophotometry in the ultraviolet-visible range (UV-VIS MSP), Raman spectroscopy, and Fourier transformed infrared spectroscopy (RS and FTIR, respectively), are the main spectroscopic techniques used for this purpose. At the same time, technology continues improving (ATR, MIR, DRIFTS) and other spectroscopic techniques can be applied in this forensic area for textile analysis (for example, inductively coupled plasma atomic emission spectroscopy—ICP-AES or X-ray fluorescence spectroscopy–XFS [37]). There are several factors that explain the increased interest in spectroscopic techniques for the study of textile fibers. These techniques are non-destructive and require only a very small amount of sample to develop an analysis. Moreover, they involve easy sample preparation and allow on-site and quick microscopic exams in a short time. Since the laser beam is around 0.8 micrometers, it can even be applied for the measurement of microfibers presented in the current market [38].

The use of spectroscopic techniques for the forensic analysis of textile fibers was presented by Pablo Prego Meleiro and Carmen García-Ruiz in their review paper from 2016 [38]. Nevertheless, a limited number of publications concerning the analysis of colored fibers dyed using disperse dyes was found in the literature [2,4,10,12].

The study of polyester fibers dyed with ternary mixtures of disperse dyes in small mass concentrations, as well as the analysis of the disperse dyes alone using spectroscopic techniques (RS and UV-VIS MSP) was performed [2]. Three types of excitation sources, 514, 633, and 785 nm, were used during RS examinations, while the UV-VIS MSP study was conducted in the 200 to 800 nm range. Analyzing the obtained results, it was observed that the He–Ne laser (k = 633 nm) turned out to be the least effective in measurements of the Raman spectra of disperse dyes (no spectrum for 50% of samples). Application of a 785 nm line of excitation allowed for an increase in the amount of interpretable Raman spectra to 75%. Using a 514 nm wavelength laser for measurements made it possible to obtain the Raman spectra of all of the disperse dyes examined, in which 42% were good quality. Obtained results led to the conclusion that none of the lasers used was ideal for the study of disperse dyes, but in spite of that the majority of them were differentiated using RS [2].

The same parameters of RS were used for the analysis of fibers from the polyester fabrics dyed with ternary mixtures of disperse dyes [2]. Analysis showed that a wavelength of 633 nm was completely ineffective for these samples because no spectra were obtained. The application of a wavelength of 514 nm produced good quality spectra for 50% of the samples but a wavelength of 785 nm was necessary to achieve good quality spectra for all polyester fiber samples. Spectra for dyed and undyed polyester fibers and spectra for dyes used were evaluated in terms of estimating the possibility of identifying each dye from the ternary mixture. An analysis of the results for a wavelength of 785 nm showed that bands from fiber-forming polymer (PET) were dominant in the spectra of dyed fibers. From all of the dyes, only one at the highest concentration could be identified (level of dye concentration in the dye bath amounted to 0.18%). In the same case, when wavelengths of 514 nm and 633 nm were used, good quality spectra were not obtained, mainly caused by the fluorescence [2].

Results of the study of polyester fibers carried out using UV-Vis MSP indicated strong absorption of ultraviolet light by the fiber-forming polymer. Those results led to the conclusion that this type of testing should first be carried out in the range of visible light and ultraviolet A radiation (above 310 nm). The UV-Vis MSP method also showed limited possibilities for discriminatory analysis of slightly dyed polyester fibers dyed with a mixture of disperse dyes.

The authors demonstrated that the capabilities for the discernment of polyester fibers dyed with ternary mixtures of disperse dyes were similar for both the spectroscopic methods employed—RS and UV-Vis MSP [2].

Today, UV-Vis MSP remains the most efficient method and the recommended analytical technique for the routine study of textile fibers in the forensic field [2,3,4,24]. It is the most used and the most discriminating spectroscopic technique available for the study of textile fibers, being an indispensable method for the objective evaluation and comparison of fiber colors. It should be also underlined that RS has become a good new tool in forensic studies of textile fibers, with a clear high potential to detect and identify dyes in fibers. On the other hand, FTIR spectroscopy shows a greater capacity for studying polymeric substrates compared with the study of dyes and is the recommended method for the identification of the classes and subclasses of the polymeric matrix. Co-monomers, solvents, and additives can be identified using FTIR, which allows further discrimination between the subclasses, for example of acrylic fibers [2,4,38].

In recent years, developments in Raman instrumentation, particularly regarding the juxtaposition of a spectrometer and optical microscope, have made this technique more attractive for analytical use. RS has been deemed the only technique through which molecular structural information concerning the dye present in a fiber may be obtained in a virtually nondestructive manner [2,4]. RS and FTIR spectroscopy are complementary techniques and a frequent recommendation, in order to obtain a comprehensive analysis of textile fibers for forensic purposes, is a combination of both techniques. On the other hand, new and informative analytical techniques are emerging for the analysis of textile fibers as microtraces related with a crime scene, but their application in the analysis of colored polyester fibers dyed using disperse dyes has not yet been presented.

## 3. Comparison of Chromatographic Methods for the Identification of Dyed Textile Fibers for Forensic Purposes with a Spectroscopic Approach

Both chromatographic (often associated with mass spectrometric detection) and spectroscopic techniques are used in forensic examination for the identification of dyed textile fibers.

Chromatographic techniques are destructive; however, they can give detailed information about the dyes of textile fibers. The identification of dyes is based not only on retention times but often on mass spectra or the selected, characterized *m*/*z* values of dyes. This method creates an opportunity for a comparison between dyes from the evidence and competitive dyes. It is also possible to determine the chemical structure of the dyes.

Spectroscopic techniques are non-destructive but give spectra including information derived from fiber polymers, dyes, or fiber polymers and dyes. The obtained data depend on the applied spectroscopic technique (RS, UV-Vis MSP, or FTIR) and measurement conditions. It should be emphasized that the mentioned methods are complementary and none of them are perfect.

The time of preparation of a sample and the time of its analysis is longer using chromatographic than spectroscopic techniques. In chromatographic techniques, the preparation of a sample requires the use of additional reagents and some special equipment, but the parameters of the analysis of individual types of dyes are constant. The identification of dyes is based on chromatographic (retention times) and mass spectrometric (*m*/*z* values) data.

In spectroscopic techniques, the fiber is treated as a sample but the analysis conditions must be determined individually.

In the forensic laboratory, microtraces of dyed textile fibers are examined usually by optical and sometimes electronic microscopy. Optical microscopy allows the identification of textile fibers based on the characteristics of their physicochemical structure. Optical research is concentrated mainly on stereoscopic microscopy with reflected light, biological microscopy with transmitted light, polarized light microscopy, UV light, and fluorescence microscopy. In this part of the examination, a scanning electron microscope can be used, especially to study damage to textile fibers.

It should be noted that the first step of studies on microtraces of dyed textile fibers is always the same. After the selection of samples and testing by optical microscopy, further methods have to be chosen for research, and this usually depends on the research laboratory equipment.

As mentioned, both chromatography and spectroscopy methods have advantages and disadvantages regarding the identification and comparison of dyed textile fibers. Therefore, the further choice of experimental methods depends on many reasons, e.g., type, form, and volume of evidence and equipment of the research lab.

## 4. New Challenges in Identifying Dyed Fibers for Forensic Purposes

Nowadays, there are many new challenges for scientists involved in the analysis and identification of textile fibers. They are directly related to the technological progress that is taking place in the modern world, and consequently, the creation of new materials and dyes for their coloring. These trends have been described, among others, by Tatsuya Hongu and Glyn O. Phillips in the book entitled “New Millennium Fibers” [39]. The authors classify newly formed fibers as “*superfibers*” or “*new frontier fibers*”. Their development is determined by the desire of the world to achieve two objectives. The first is to improve human health and to increase the comfort of life. The second is to improve the quality of medical care and to emphasize the use of nanotechnology in the creation of various nano-fibers. Furthermore, the increase in the share of renewable raw materials, derived from agro-culture, in the synthesis of polymers presents new challenges [39]. The fibers produced from these materials will have a different chemical composition than the fibers produced from petroleum compounds, which could potentially influence the analysis and identification of the fibers. Designing alternative, fast, and relatively inexpensive methods, using the analytical techniques available in forensic labs, will be extremely valuable for new analytical problems in this area [11].

At present, however, the old methods are usually used during the analysis of fibers secured as evidential material at crime scenes. Microscopy dominates this field by offering a wide range of analyses. In combination with other analytical techniques, it is the most important tool for fiber identification and comparison. Microscopy should be the first choice for any fiber researcher.

However, it is important to use and optimize new and more informative analytical techniques for the analysis of textile fibers as traces related with crime scenes, such as infrared chemical imaging spectroscopy, X-ray fluorescence, and metal underlayer ATR spectroscopy (MU-ATR), as well as chromatographic techniques with different detection systems [11,12,13,14,15,16,17,18,19,20,21,40].

Any kind of damage sustained by a textile product through use, lowering its aesthetic and practical value, is disadvantageous from the user’s as well as the producer’s perspective. However, forensic material that has some sort of characteristic damage can have greater evidential value. For this reason, research into the destructive processes that act on a textile product in the course of its use, or damage due to the effects of specific chemical and physical factors, is important from a forensic standpoint as well, since it can help in the process of ascertaining the course of events and circumstances of a crime [40]. This is why it is important to continue research into new destructive agents that give each textile fiber its individual character.

## 5. Conclusions

The discrimination of fiber samples is crucial for forensic scientists throughout the world and the research methods in this field should be developed in order to minimize the possibility of mistakes. This review sheds new light on both suspension (disperse) dyes and dyed polyester fibers, and their identification using chromatographic techniques with different detection systems. The presentation of chromatographic procedures, and the comparison of their usefulness with spectroscopic approaches, allows the identification of which analytical method is best suited for the effective characterization of polyester fibers dyed with disperse dyes. This knowledge could help formulate the correct forensic expertise without the risk of mistakes.

## Supplementary Materials

The following are available online, Tables S1–S3. Table S1: Overview of the extraction procedures of disperse dyes from dyed polyester fibers; Table S2: Overview of chromatographic methods described in the literature [12,13,14,15,16,17,18,19,20,21] for the identification of disperse dyes extracted from polyester fibers (chromatographic conditions); Table S3: Overview of chromatographic methods described in the literature [12,13,14,15,16,17,18,19,20,21] for the identification of disperse dyes extracted from polyester fibers (selected qualification and quantification parameters).
