# Supplementary material for: The Identification of Polyester Fibers Dyed with Disperse Dyes for Forensic Purposes
Source: Molecules. 2019 Feb 10;24(3):613. doi: 10.3390/molecules24030613 (PMC6384617; doi:10.3390/molecules24030613)
Supplement: Supplementary file 1 [file molecules-24-00613-s001.pdf]

molecules

MDPI

Appendix

Review

The identification of polyester fibers dyed with disperse dyes for forensic purposes

Daria Śmigiel-Kamińska[1*], Jan Pośpiech[1], Joanna Makowska[1], Piotr Stepnowski[1], Jolanta Wąs-Gubała[2], Jolanta Kumirska[1*]

[1] Faculty of Chemistry, University of Gdańsk, ul. Wita Stwosza 63, 80-308 Gdansk, Poland
[2] Institute of Forensic Research, Criminalistics Department, Westerplatte 9, 3-033 Krakow, Poland
* Correspondence: d.smigiel-kaminska@pgdstud.edu.da.pl; jolanta.kumirska@ug.edu.pl; Tel.: +48 58 523 52 12

Received: date; Accepted: date; Published: date

Tables content:

Table 1A. Overview of the extraction procedures of disperse dyes from dyed polyester fibers

Table 2A. Overview of chromatographic methods described in the literature [12-21] for the identification of disperse dyes extracted from polyester fibers (chromatographic conditions)

Table 3A. Overview of chromatographic methods described in the literature **[12-21]** for the identification of disperse dyes extracted from polyester fibers (selected qualification and quantification parameters).

molecules MDPI

Table S1. Overview of the extraction procedures of disperse dyes from dyed polyester fibers.

No. method	Dyes C.I. name (dye dispersol name)	Solvents	Fiber Length	Volume	Temperature	Time	Lit.
1	Disperse Red 54 (Scarlet CG)<br>Disperse Blue 122 (Navy D-2G-133)<br>Mix (Black BT)<br>Disperse Orange 13 (Orange CB)<br>Mix (Black D-2B)<br>Disperse Yellow 60 (Yellow D-3R)<br>Disperse Yellow 54 (Yellow C-4R)<br>Disperse Red 74 (Scarlet BR)<br>Disperse Violet 33 (Rubine C-B)<br>Disperse Red 282 (Red D-2G)<br>Disperse Red 82 (Red C-3B)<br>Disperse Blue 29 (Navy C-4R)<br>Disperse Yellow 126 (Yellow D-7G)<br>Disperse Yellow 119 (Yellow C-5G)<br>Disperse Yellow 24 (Yellow CT)<br>Disperse Yellow 1 (Yellow BA)	The best solvent: boiling chlorobenzene	Fibers or small pieces of fabrics	0.5 – 3 mL	Boiling point solvent	5 min or until most of the dye has been extracted	[12]
2	Disperse Violet 8 (Violet C-BR)<br>Disperse Yellow 1 (Yellow B-A)<br>Disperse Red 11 (Red B-3B)<br>Disperse Yellow 126 (Yellow D-7G)<br>Disperse Blue 26:1 (Blue B-2G)<br>Mix (Black D-2G)	Chlorobenzene<br><br>Dimethylformamide-acetonitrile (1: 1, v/v)	2 - 5 mm	5 μL<br><br>4 μL	100°C<br><br>120 - 130°C	15 min<br><br>1 - 2 min	[13]

	Disperse Brown 4 (Brown D-3R)<br>Disperse Blue 83 (Blue D-2R)<br>Disperse Yellow 42 (Yellow C-T)<br>Disperse Yellow 119 (Yellow C-5G)<br>Disperse Blue 35 (Navy B-T)<br>Disperse Red 82 (Red C-3B)<br>Disperse Yellow 39 (Yellow B-GR)<br>Disperse Red 91 (Red C-B)<br>Disperse Red 54:1 (Scarlet C-2R)<br>Disperse Red 282 (Red D-2G)<br>Disperse Violet 33 (Rubine C-B)<br>Mix (Black D-R)<br>Mix (Black D-B)<br>- (Navy D-4G)<br>Disperse Red 54 (Scarlet C-G)<br>Disperse Orange 30 (Orange C-R)<br>- (Navy C-R)<br>Mix (Black D-2B)<br>Disperse Orange 25 (Orange B-2R)<br>- (Navy C-2R)<br>Disperse Blue 122 (Navy D-2G)<br>Disperse Blue 295 (Blue C-3G)<br>Disperse Blue 79:1 (Navy D-3GR)<br>- (Red C-G)<br>Disperse Red 131 (Red D-B)<br>Disperse Red 158 (Scarlet B-RN)<br>Disperse Blue 296 (Blue D-6G)	*Dimethyloformamide was modified by adding 2,6-di-*tert*-butyl-4-methylphenol and citric acid					

	Disperse Blue 87 (Turquoise C-G)<br>Disperse Blue 185 (Turquoise C-R)<br>Disperse Blue 56 (Blue B-R)<br>Disperse Brown 19 (Brown C-3G)<br>- (Brown C-CM)<br>Disperse Orange 32 (Orange D-G)<br>Disperse Orange 54 (Yellow C-4R)<br>Disperse Blue 200 (Blue D-4G)<br>Mix (Black B-T)<br>Disperse Yellow 218 (Yellow B-6G)<br>Disperse Green 9 (Green C-6B)<br>Disperse Red 60 (Red B-2B)<br>Disperse Violet 63 (Violet C-3R)<br>Disperse Blue 29 (Navy C-4R)<br>Mix (Black C-3G)<br>Mix (Black C-MD)<br>- (Navy C-2G)<br>Disperse Yellow 183 (Yellow D-8G)<br>Disperse Yellow 54 (Yellow C-3G)<br>- (Orange C2-G)<br>Disperse Orange 13 (Orange C-B)<br>Disperse Orange 60 (Yellow D-3R)<br>- (Rubine B-B)<br>Disperse Blue 72 (Violet B-G)						
**3**	Disperse Yellow 82<br>Disperse Violet 95<br>Disperse Orange 45	Chlorobenzene	10 mm	5 µL	130 °C	30 min	**[14]**

	Disperse Yellow 211<br>Disperse Blue 125<br>Disperse Red 151<br>Disperse Violet 57<br>Disperse Blue 165<br>Disperse Red 349						
**4**	Disperse Blue 73	DMSO	10 mm	20 µL	100 °C	< 2 h	**[15]**
**5**	Disperse Violet 77<br>Disperse Blue 60<br>Disperse Yellow 114	Chlorobenzene	0.5; 2; 5 mm	100 µL	100 °C	1 h	**[16]**
**6**	Disperse Blue 60<br>Disperse Blue 291<br>Disperse Blue 366<br>Disperse Red 73<br>Disperse Red 145<br>Disperse Red 323<br>Disperse Orange 30<br>Disperse Yellow 82<br>Disperse Yellow 163	ACN/water (4:3, v/v)<br>MeOH/water (1:1, v/v)<br>DMF	5 mm<br>7 mm (thread)	30 µL	100 °C	30 min	**[17]**
**7**	Disperse Red 4	Chlorobenzene	20 mm	20 µL	130 °C	10 min	**[18]**
**8**	Disperse Orange 3<br>Disperse Red 11<br>Disperse Yellow 3<br>Disperse Yellow 9<br>Disperse Yellow 39<br>Disperse Blue 3	MeOH	0.5 g cut fabric	2 x 5 mL	70 °C<br>ultrasonification	2 x 15 min	**[19]**

	Disperse Red 1<br>Disperse Orange 1<br>Disperse Blue 106<br>Disperse Red 17<br>Disperse Blue 102<br>Disperse Yellow 49<br>Disperse Blue 124<br>Disperse Orange 37<br>Disperse Brown 1<br>Disperse Yellow 1<br>Disperse Blue 1						
**9**	Disperse Red 167<br>Disperse Red 60<br>Disperse Red 153<br>Disperse Yellow 211<br>Disperse Orange 288<br>Disperse Red 82<br>Disperse Orange 31<br>Disperse Violet 93<br>Disperse Blue 291G	DMSO	10 mm	20 μL	100 °C	Discoloured or to a maximum 2 h	[20]
**10**	Disperse Yellow 9<br>Disperse Red 1<br>Disperse Violet 26<br>Disperse Brilliant Red SF-B	Acetonitrile	a 0.5 cm piece of polyester fiber	20 μL	60 °C	Ultrasonication for 5 min followed by heating in a water bath at 60 °C for 60 min	[21]

Table S2. Overview of chromatographic methods described in the literature [12-21] for the identification of disperse dyes extracted from polyester fibers (chromatographic conditions)

No. method	Analytes	Technique	Column	The mobile phase	Gradient program	The mobile phase flow rate	Injection	Lit.
1	Table.1 No. 1	HPLC	250 mm x 3.2 mm column packed with 10 μm LiCrosorb Si-60<br><br>Pre-column 40 mm long packed with silica 60, particle size of about 400 μm	A - hexane<br>B - ethyl acetate	25 - 100% B (0 - 20 min)<br>100% B (20 - 24 min)<br>than automatically reset to 25% B	1 mL/min	10 μL	[12]
2	Table 1 No. 2	HPLC	125 mm, 3 mm I.D., 6.3 mm O.D. stainless steel column packed with 5 μm Hypersil ODS (Shandon Southern Cheshire, U.K.)	Acetonitrile : water (4:1, v/v) was buffered to pH 3.2 by the addition of 1 g of citric acid per litre	-	0.4 mL/min	-	[13]
3	Table 1 No. 3	HPLC	150 x 2.1 mm I.D., 5 μm particle size Intersil ODS-2 (GL Sciences Inc., Tokyo, Japan).	A - water<br>B - acetronitrile<br>Both eluents had pH 2.5 by the addition of 1 mL per litre of 2,5 M sulphuric acid	20 - 98% B (0 - 15 min)<br>98 - 20% B (15 - 20 min)	0.5 mL/min	5 μL	[14]
4	Table 1 No. 1	HPLC	Grom-sil 120 ODS-5 ST (3 μm, 2 x 150 mm) Grace Davison Discovery Sciences, Deerfield, USA	A – 10 mM ammonium acetate in water : MeOH (95:5, v/v)<br>B – 25 mM ammonium	50 % B (0 - 53 min)<br>100 % B (53 - 67 min)	n. d.	10 μL	[15]

				acetate in ACN : MeOH (50: 50, v/v)				
**5**	Table 1 No. 2	UPLC	BEH C18 column (1.7 µm , 2.1 x 50 mm)<br>Waters Acquity UPLC®	A - MeOH, 0,15 % formic acid<br>B - 50 mM ammonium acetate	UPLC-DAD<br>90 % B (0 - 0.5 min)<br>10 % B (0.5 - 4 min)<br>10 %B (4 - 4.5 min)<br>90 % B (4.5 - 4.6 min)<br>90%B(4.6-6.5min)<br>UPLC-MS/MS<br>80 % B (0 - 0.5 min)<br>20 % B (0.5 - 4 min)<br>20 %B (4 - 4.5 min)<br>80 % B (4.5 - 4.6 min)<br>80 % B (4.6 - 6.5 min)	UPLC - DAD<br>0.6 mL/min<br>UPLC – MS/MS<br>0.3 mL/min	10 µL	**[16]**
**6**	Table 1 No. 3	HPLC	2 ODS (5 µm, 1.5 x 150 mm)<br>Chemicals Evaluation and Research Institute, Japan	A - 5 % MeOH, 10 mM ammonium acetate<br>B - 95 % MeOH, 10 mM ammonium acetate	60 – 100 % B (0 - 15 min),<br>100 % B (15 - 25 min)	0.2 mL/min	5 µL	**[17]**
**7**	Table 1 No. 4	HPLC	ZORBAX Eclipse XDB-C18 (2.1 x 150 mm)	A - ACN<br>B - H_2O	70 % B (0 - 5 min)<br>90 % B (5 - 25 min)<br>90 % B (25 - 40 min)	0.2 mL/min	5 µL	**[18]**
**8**	Table 1 No. 5	UHPSFC	- Waters ACQUITY UPC2™ BEH (1.7 µm, 3 x 100 mm),<br>- Waters ACQUITY UPC2™ BEH 2-Ethyl-pyridine (1.7 µm, 3 x 100 mm)	A - MeOH<br>B - CO_2	1 – 3 % B (0 - 1.5 min),<br>3 – 7 % B (1.5 - 2 min),<br>7 – 10 % B (2 - 3 min),<br>10 – 15 % B (3 - 3.3 min),	2 mL/min	1 µL	**[19]**

			- Waters ACQUITY UPC2™ HSS C18 SB (1.8 µm, 3 x 100 mm)<br>- Waters ACQUITY UPC2™ CSH Fluorophenyl (1.7 µm, 3 x 100 mm)		15 – 16 % B (3.3 - 4.0 min),<br>16 – 20 % B (4.0 - 4.5 min)			
**9**	Table 1 No. 9	HPLC	Grom-sil 120 ODS-5 ST (150 x 2.0 mm i.d., 3 µm)<br>Grace Davison Discovery Sciences, Deerfield, USA	A – 10 mM ammonium acetate in water : MeOH (95:5, v/v)<br>B – 25 mM ammonium acetate in ACN : MeOH (50:50, v/v)	-	n. d.	10 µL	**[20]**
**10**	Table 1 No. 10	HPLC-MS/MS	Symmetry C18 (50 mm × 1.0 mm I.D., 3.5 µm,<br>Ireland)	(A) 0.1% Acetic acid in water<br>(B) 0.1% Acetic acid in acetonitrile	From 95:5 A:B (v/v) to 24:76 A:B (v/v)A (0 - 15 min),<br>100% B (for 0.1 min and retention for 2 min),<br>95:5 A:B (v/v) (for 0.1 min and retention for 2 min)	0.3 mL/min	10 µL	**[21]**

Table S3. Overview of chromatographic methods described in the literature **[12-21]** for the identification of disperse dyes extracted from polyester fibers (selected qualification and quantification parameters).

No.	Analytes	Technique	Detector	Scanned wavelength range	Retention time	[m/z]	LOD	Lit.
1	Table.1 No. 1	HPLC	UV-VIS	600, 500, 420 nm	Between 1.09 and 7.78 min	-	-	[12]
2	Table 1 No. 2	HPLC	Single wavelength detection: UV-VIS<br>Multiple wavelength detection: Linear diode array (LDA)	400, 500, 600 nm<br>250 - 600 nm	Relative retention times with respect to 2,6-di-*tetr*-butyl-4-methylphenol (t_R = 7.0 min) (0.27 to 1.59)	-	200 pg	[13]
3	Table 1 No. 3	HPLC	Photodiode-array (PDA)	380 - 600 nm	between 18.0 and 22.0 min<br>Relative retention times with respect to Rhodamine B (0.859 to 1.355)	-	80 - 1280 pg	[14]
4	Table 1 No. 1	HPLC	Diode array detection (DAD)<br>MS(ESI)-LTQ Orbitrap (HRMS)	200 - 800 nm (λ_{max} 627 nm)<br>150 – 2000 m/z	45.6 min<br>51.0 min	Calculated mass [m/z]<br>361.08190<br>375.09755	DAD – 50.7 μg/L powder/ 0.14 mm fiber<br>HRMS – 1.1 μg/L powder/0.003 mm fiber	[15]
5	Table 1 No. 2	UPLC	DAD	325 - 675 nm	between 2.5 and 5.0 min	441→221<br>424→348	DAD – 10.10 to 37.00 ppb	[16]

			MS(ESI) tandem quadrupole mass spectrometer<br>MS/MS - Selected Reaction Monitoring (SRM) mode		total runtime of 6.5 minutes	425→178	MS/MS - 0.62 - 4.67 ppb	
**6**	Table 1 No. 3	HPLC	DAD<br>MS(ESI) linear ion trap (LIT) tandem MS<br>MS/MS - SRM mode	380 - 760 nm	total runtime of 50 minutes<br>e.g. Disperse Red 145 t_R = 9.07 min	380→348<br>509→480<br>363→176<br>349→308<br>381→73<br>534→444<br>450→390<br>334→290<br>417→376	DAD -750 to 1750 pg<br>LIT-MS/MS (SRM) - 1 do 15 pg	**[17]**
7	Table 1 No. 4	HPLC	DAD<br>MS(ESI) quadrupole mass spectrometer<br>MS - Single Ion Monitoring (SIM) mode	200 - 700 nm	n.d.<br>total runtime of 40 minutes	Calculated mass [m/z]<br>270	n. d.	**[18]**
**8**	Table 1 No. 5	UHPSFC	MS(ESI) triple quadrupole tandem MS<br>MS/MS - Multi Reaction Monitoring (MRM) mode	n. d.	between 2 and 4.5 min	Parent /quantitative/qualitative daughter ions [m/z]<br>243.2 / 121.9 / 91.9<br>269.3 / 254.0 / 226.2<br>270.3 / 107.0 / 150.0<br>275.3 / 228.2 / 258.2<br>291.3 / 129.9 / 245.1	MS/MS - 0.02–1 μg/mL	**[19]**

						297.2 / 252.1 / 235.1<br>315.3 / 134.6 / 255.2<br>319.2 / 169.1 / 121.9<br>336.3 / 178.1 / 70.0<br>345.3 / 164.1 / 177.1<br>366.3 / 208.1 / 147.1<br>375.3 / 238.2 / 164.0<br>378.3 / 86.9 / 220.2<br>392.2 / 351.2 / 323.1<br>433.2 / 197.0 / 185.1<br>276.3 / 229.1 / 259.1<br>269.2 / 106.8 / 253.2		
**9**	Table 1<br>No. 9	HPLC	DAD<br>MS(ESI)-LTQ Orbitrap (HRMS)	200 - 800 nm<br>150 – 2000 m/z	50.1 min<br>43.8 min / 53.3 min<br>53.5 min<br>46.6 min<br>49.6 min<br>45.9 min<br>49.6 min<br>52.0 min<br>54.1 min	504.1289<br>392.0594 / 376.0640<br>462.0564<br>360.0505<br>444.1677<br>498.1627<br>444.1677<br>434.1108<br>532.071	-	**[20]**
**10**	Table 1<br>No. 10	HPLC-<br>MS/MS	MS(ESI) quadrupled type<br>tandem MS<br>MS/MS - MRM mode	n.d.	5.20 min<br>9.35 min	273.0→225.85<br>315.0→134.05<br>315.0→255.05	MS/MS – 0.01-1.00<br>ng/mL	**[21]**

					12.20 min	423.2→346.05<br>423.2→405.25		
					10.58 min	411.1→332.25<br>411.1→163.1		

References (position identical as in the manuscript)

12. West, J.C. Extraction and analysis of disperse dyes on polyester textiles, *J. Chromatogr*. **1981**, *208*, 4754.
13. Wheals, B.B.; White, P.C.; Paterson, M.D. High-performance liquid chromatographic method utilising single or multi-wavelength detection for the comparison of disperse dyes extracted from polyester fibers, *J. Chromatogr*. **1985**, *350*, 205-2015.
14. Speers, S.J.; Little, B.H.; Roy, M. Separation of acid, basic and dispersed dyes by a single-gradient elution reversed-phase high-performance liquid chromatography system, *J. Chromatogr. A* **1994**, *674*, 263 - 270.
15. Carey, A.; Rodewijk, N.; Xu X.; Van Der Weerd, J. Identification of dyes on single textile fibers by HPLC-DAD-MS, *Anal. Chem.*, **2013**, *85*, 11335–11343, DOI: 10.1021/ac402173e.
16. J. Hoy, S. J. Development and Figures of Merit of Microextraction and Ultra-Performance Liquid Chromatography for Forensic Characterization of Dye Profiles on Trace Acrylic, Nylon, Polyester, and Cotton Textile Fibers", *doctoral dissertation*, University od South Carolina, Columbia, 2013.
17. Kato T.; Suzuki Y.; Handa M.; Extraction and Analysis of Disperse Dyes from Colored Polyester Single Fibers Using Liquid Chromatography/Linear Ion Trap Tandem Mass Spectrometry, *Anal. Sci.* **2016**, *32*, 1019–1022, DOI: 10.2116/analsci.32.1019.
18. Dorrien, D. M. Discrimination of Automobile Carpet Fibers Using Various Analytical Techniques and the Subsequent Creation of a Comprehensive Database, *doctoral dissertation*, University of Central Florida, Orlando, 2006.
19. Zhou, Y.; Du, Z.; Zhang, Y.; Simultaneous determination of 17 disperse dyes in textile by ultra-high performance supercritical fluid chromatography combined with tandem mass spectrometry, *Talanta* **2014**, *127*, 108–115, DOI: 10.1016/j.talanta.2014.03.055.
20. Schotman, T. G.; Xiu, X.; Rodewijk, N.; van der Weerd, J.; Application of dye analysis in forensic fibre and textile examination: Case examples, *Forensic Sci. Int.*, **2017**, *278*, 338-350, DOI 10.1016/j.forsciint.2017.07.026.
21. Hu C.; Zhu, J.; Mei, H.; Shi, H.; Guo, H.; Zhang, G.; Wang, P.; Lu, L.; Zheng, X. A sensitive HPLC-MS/MS method for the analysis of fiber dyes. *Forensic Chem.*, **2018**, *11*, 1-6, DOI: 10.1016/j.forc.2018.08.001

© 2018 by the authors. Submitted for possible open access publication under the terms and conditions of the Creative Commons Attribution (CC BY) license (http://creativecommons.org/licenses/by/4.0/).
